# Reliability of ultrasound imaging in the assessment of the dorsal Lisfranc ligament

**DOI:** 10.1186/1757-1146-6-7

**Published:** 2013-03-03

**Authors:** David D Rettedal, Nathan C Graves, Joshua J Marshall, Katherine Frush, Vassilios Vardaxis

**Affiliations:** 1 College of Podiatric Medicine and Surgery, Des Moines University, Des Moines, IA, USA; 2 Department of Physical Therapy, Des Moines University, Des Moines, IA, USA; 3 Department of Orthopaedic Surgery, Division of Podiatry, Shands/University of Florida, Jacksonville, FL, USA

**Keywords:** Reliability, Ultrasound, Lisfranc ligament

## Abstract

**Background:**

The Lisfranc ligament plays an integral role in providing stability to the midfoot. Variable clinical presentations and radiographic findings make injuries to the Lisfranc ligament notoriously difficult to diagnose. Currently, radiographic evaluation is the mainstay in imaging such injuries; however, ultrasound has been suggested as a viable alternative. The objective of this study was to evaluate the intra-rater and inter-rater reliability in the measurement of the length of the dorsal Lisfranc ligament using ultrasound imaging in healthy, asymptomatic subjects.

**Methods:**

The dorsal Lisfranc ligaments of fifty asymptomatic subjects (n = 100 feet) were imaged using a Siemens SONOLINE Antares Ultrasound Imaging System^©^ under low, medium, and high stress loads at 0° and 15° abducted foot positions. The lengths of the ligaments were measured, and Interclass correlation coefficients were used to calculate within-session intra-rater reliability (n = 100 feet) as well as between-session intra-rater reliability (n = 40 feet) and between-session inter-rater reliability (n = 40 feet).

**Results:**

The within-session intra-rater reliability results for dorsal Lisfranc ligament length had an average ICC of 0.889 (min 0.873 max 0.913). The average ICC for between-session intra-rater reliability was 0.747 (min 0.607 max 0.811). The average ICC for between-session inter-rater reliability was 0.685 (min 0.638 max 0.776).

**Conclusions:**

The measurement of the dorsal Lisfranc ligament length using ultrasound imaging shows substantial to almost perfect reliability when evaluating asymptomatic subjects. This imaging modality methodology shows promise and lays the foundation for further work in technique development towards the diagnostic identification of pathology within the Lisfranc ligament complex.

## Background

**Figure 1:**
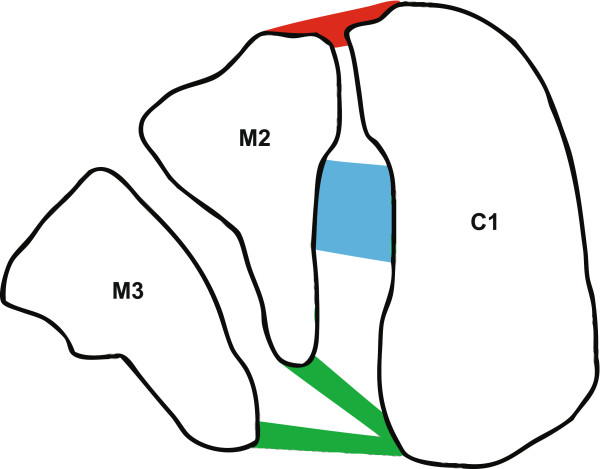
**Cross-sectional illustration through the Lisfranc joint.** C1 = Medial cuneiform, M2 = 2^nd^ metatarsal base, M3 = 3^rd^ metatarsal base, Red = dorsal Lisfranc ligament, Blue = interosseous Lisfranc ligament, Green = plantar Lisfranc ligament.

Injuries to the Lisfranc complex account for 0.2% of all orthopedic injuries [1]. This rare injury has a frequency of approximately 1 case per 55,000 persons each year [2]. Lisfranc complex injuries are most commonly high-energy injuries that occur when an axial load or rotational force is brought on a foot fixed in a plantar-flexed position [3]. Often males in the third decade of life sustain such an injury as a result of a fall from a height, a motor vehicle accident, or a sporting injury [3]. Despite its low frequency, around 20% of initially presenting Lisfranc joint injuries in emergency departments are either unrecognized or misdiagnosed [4], suggesting that the current evaluation methods of midfoot pain are unreliable. It is crucial to find a technique that consistently evaluates midfoot pain because injury to the Lisfranc joint can have potentially long-term complications when treatment is inadequate, inappropriate, or delayed due to an initially missed diagnosis. Complications include chronic pain, arthritis, and functional loss due to ligamentous injury [5]. The Lisfranc ligament complex, which spans between the medial cuneiform and base of the second metatarsal, is critical in providing stability to the midfoot [6]. The ligament complex is divided into three distinct segments, dorsal, interosseous, and plantar (Figure 1). The interosseous segment is the largest and strongest while the dorsal segment is the smallest and weakest [7]. Current methods of evaluating the integrity of the Lisfranc ligament complex rely heavily upon standard radiographs which have high false negative rates [8]. High false negative rates of the current diagnostic techniques demand re-evaluation. Exploring new applications of other existing, readily available modalities could lead to a more accurate diagnosis.

Care must be taken when evaluating a patient presenting with midfoot pain because Lisfranc injuries are reported to be one of the most common types of injuries in malpractice cases against radiologists and emergency physicians [5]. Meticulous history taking usually shows that patients with Lisfranc ligament complex injuries have severe pain at the time of injury, at a much higher pain rating than would be expected with an ankle or another midfoot sprain [1]. Clinical examination usually reveals dorsal midfoot swelling that is painful upon palpation, often accompanied by ecchymosis [1] and the inability to bear weight [9].

Currently accepted diagnostic guidelines state that Lisfranc ligamentous instability is seen with greater than 2 mm of diastasis between the medial cuneiform and the base of the second metatarsal as seen on standard radiographs [10]. The most accepted diagnostic technique is weight-bearing radiographs, compared to the contralateral side [11]. Manual stress radiographs can also be used to detect diastasis, but stress views can be quite painful and a significant level of anesthesia must be obtained [1]. Weight-bearing and stress radiographs do not always detect the medial cuneiform-second metatarsal diastasis. There are variable radiographic findings with variations in foot supination-pronation and with orientation of the x-ray beam [12]. Furthermore, Nunley et al. found that low energy sprains to the Lisfranc ligament complex could produce enough pain to limit athletes from playing sports but show a completely undisplaced joint on weight-bearing radiographs [13]. In a recent study Raikin et al. assessed the use of magnetic resonance imaging for diagnosis of Lisfranc ligament complex injuries when compared to intraoperative findings. In their study, 90% of unstable Lisfranc complexes were correctly classified with a sensitivity, specificity, and positive predictive value of 94%, 75%, and 94% respectively [14] suggesting that direct visualization of the Lisfranc ligament complex with MRI is an accurate method for detecting instability. In their study, the plantar Lisfranc segment was the best predictor of midfoot instability.

Pure Lisfranc ligamentous injury may be present with few radiographic changes and variable physical findings [12]. Because of these shortfalls in standard radiographs, a diagnostic technique providing direct ligamentous visualization is advantageous. While only MRI has the ability to evaluate all three segments of the Lisfranc ligament [14], ultrasound has been proposed by Woodward et al. as viable diagnostic technology to view Lisfranc ligamentous injuries [6]. Using ultrasound, the integrity of the interosseous segment of the Lisfranc ligament must be inferred upon by imaging the dorsal Lisfranc ligament. This is due to the narrow medial cuneiform-second metatarsal articulation which makes sonographic visualization of the interosseous segment impossible. Their study advocates using the dorsal Lisfranc ligament as an indirect sign of interosseous Lisfranc ligament disruption [6]. They concluded that when the dorsal Lisfranc ligament is intact, the interosseous segment is likely intact as well; however, when the dorsal segment is disrupted in conjunction with an increased diastasis between the medial cuneiform and second metatarsal base, the interosseous segment of the Lisfranc ligament is likely torn [6].

When compared to MRI, ultrasound is much more portable and less time consuming. The subject can be comfortably positioned as the technician gathers imaging without the apprehension of laying in an MRI unit. Ultrasound is also considerably less expensive than MRI [15]. It gives dynamic, real-time imaging to view the structures surrounding the ligament allowing the technician to assess any other soft tissue abnormalities. This is all done without any radiation emitted toward patient or technician, like radiography [15]. Although ultrasound has distinct advantages to radiography and MRI, to our knowledge there is currently no literature to support the reliability of using ultrasound imaging to take measurements of the dorsal Lisfranc ligament. We would like to determine if ultrasound imaging measurements of the dorsal Lisfranc ligament can be acquired in a consistent manner. The aim of this study was to determine the intra-rater and inter-rater reliability of measuring the length of the dorsal Lisfranc ligament protocol in non-symptomatic young adults using diagnostic ultrasound, under both unloaded as well as under physiologically/clinically reasonable load conditions.

## Methods

### Subjects

Fifty healthy subjects for a total of one-hundred (n = 100) asymptomatic feet participated in the project. Inclusion criteria for the subjects required that they were free from current foot pain, without any musculoskeletal disabilities, and between the ages 20 to 45. Exclusion criteria included any subject who had a prior foot surgery, congenital foot abnormality, trauma to the feet within the last 10 years, allergy to ultrasound transmission gel, and currently pregnant females. Written informed consent was obtained from each test subject prior to participation in the study. Institutional review board approval was received from the Des Moines University human subjects committee before beginning the project.

### Equipment

**Figure 2:**
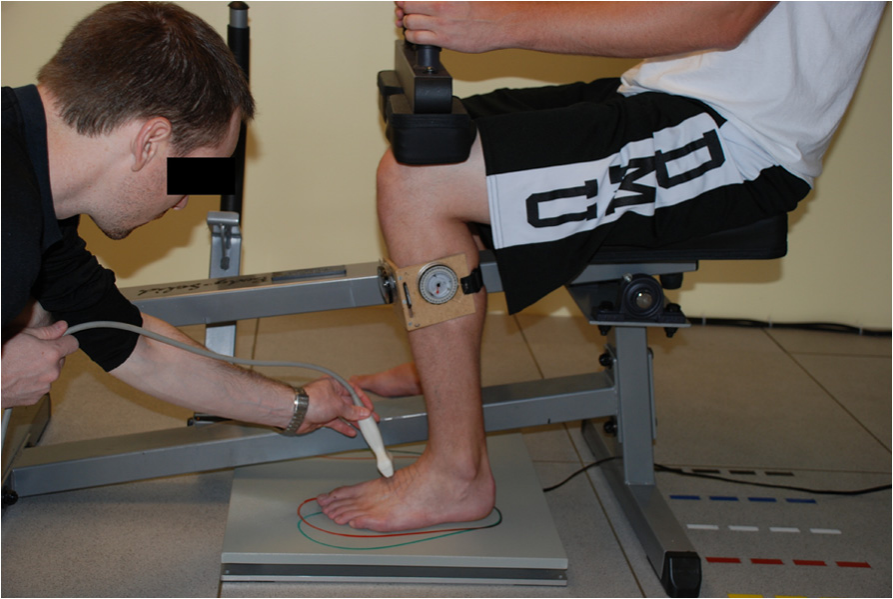
**In-house modified calf raise apparatus with force plate under the loading pad.** Subject’s knee and ankle are both flexed at 90° with the tibia perpendicular to the ground. The 0° and 15° abducted footprints can be visualized as well as the parallel lines drawn on the dorsum of the subject’s foot parallel to the Lisfranc ligament.

A commercially available calf raise apparatus (Figure 2) was purchased and was modified in-house to allow for lower leg placement directly under the knees with the tibia perpendicular to the ground and both knee and ankle joints positioned at 90° relative angles. A Siemens SONOLINE Antares Ultrasound Imaging System^©^ (Siemens Medical Solutions USA, Inc., Issaquah, WA) with a 10.0 MHz linear array transducer was used to acquire images of the dorsal Lisfranc ligament. Ultrasound images were saved in digital format for off-line measurement of the ligament length using in-house written software in MATLAB^©^ (The MathWorks, Inc., Natick, MA).

### Experimental protocol

The dorsal Lisfranc ligament was sonographically assessed under low, intermediate, and high stress loads (approximately equivalent to non weight-bearing leg weight, bipedal stance, and single leg stance respectively) at 0° and 15° abducted foot orientation positions. Stress conditions were applied using the seated calf raise apparatus loaded with weight plates. The moment arm ratio of knee compression location to weight plate load location was 16/45 and the loads used were such to achieve zero, fifty and one hundred percent of each subject’s body weight. Foot prints with 0° and 15° of abduction were used to ensure the two appropriate foot orientation positions. Three trials at each stress/foot angle condition were completed with a randomized protocol. Subjects’ feet were assessed independently of each other, resulting at 18 ultrasound images (3 trials of the 6 stress/foot angle conditions) taken for each foot during each testing session.

**Figure 3:**
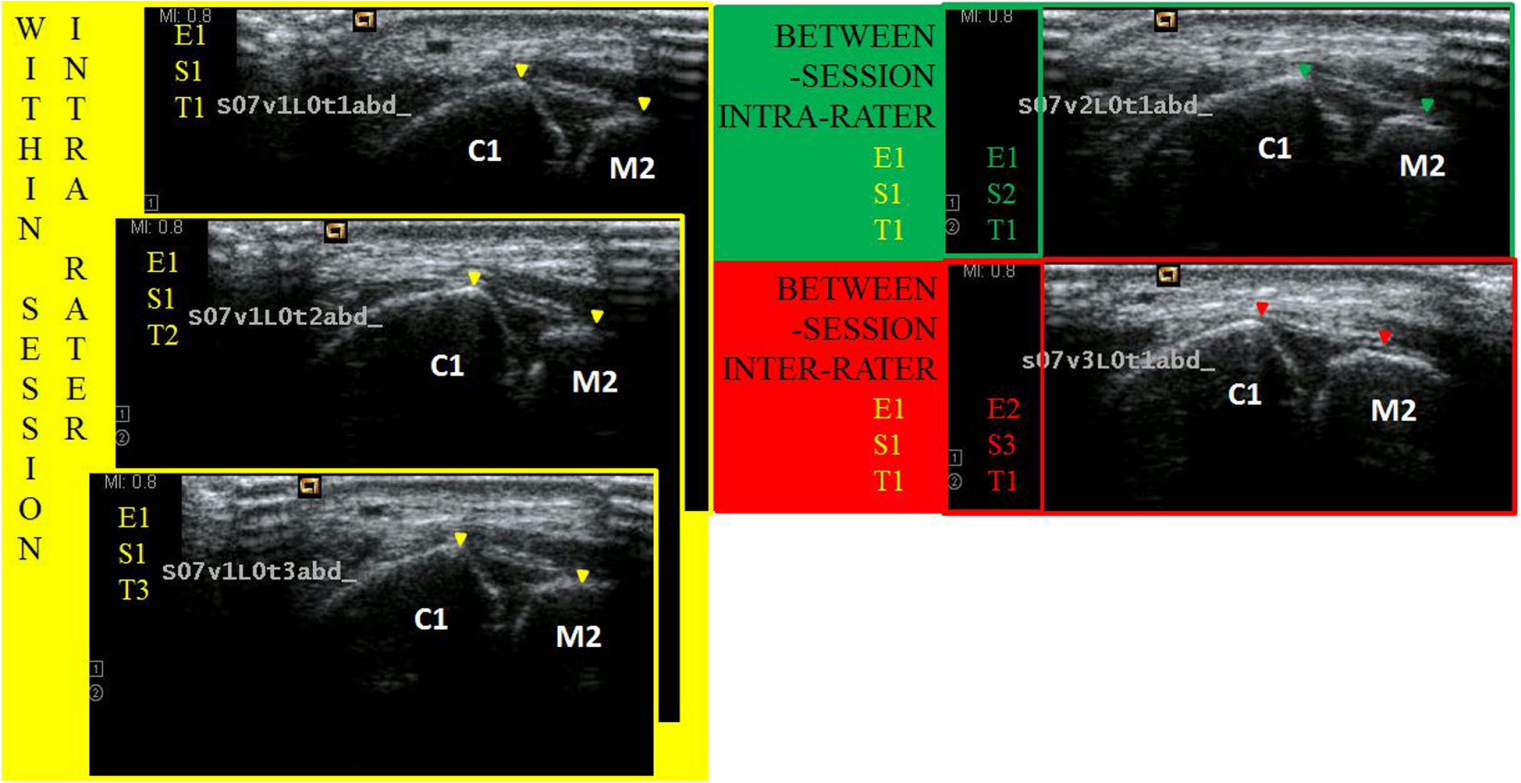
**Illustration of reliability levels using sample images from the respective conditions.** The sonogram images connected with color codes (yellow, green, and red) reflect (Within-session Intra-rater; Between-session Intra-rater; and Between-session Inter-rater, respectively) levels of reliability. The boundaries of the dorsal Lisfranc ligament are identified with the colored arrowheads on each sonogram. (E = Examiner, S = Session, T = Trial, the numbers reflect incremental levels).

The reliability of the dorsal Lisfranc ligament length measurement under each stress/foot orientation condition was determined with ICCs by using analysis of variances (ANOVA) on SPSS version 18.0. The ICC model 2 for a single measurement (ICC 2,1) was used for all reliability analyses of the dorsal Lisfranc ligament length measurements. The following illustration (Figure 3) demonstrates how reliability was determined using an exemplar stress/foot orientation condition on an example subject. Fifty subjects (n = 100 feet) reported for an initial session with Examiner A (D.D.R.). Within-session intra-rater reliability was determined comparing the 3 trials for each of the six conditions.

Twenty randomly selected subjects (n = 40 feet) were asked to return at least 24 hours later for a second session with D.D.R. performing the same ultrasound protocol. Between-session intra-rater reliability was determined comparing trial one of each condition from the initial session to trial one of the same condition from the second session with the same rater.

Another set of twenty randomly selected subjects (n = 40 feet) were asked to return at least 24 hours after their earlier session to be measured by Examiner B (J.J.M.). Both examiners were trained similarly and performed the same randomized protocol. Measurements taken from the initial session with D.D.R. and the session with J.J.M. were compared to determine between-session inter-rater reliability, using the first trial of the corresponding conditions. The ultrasound examiners, D.D.R. and J.J.M., had no formal ultrasound training prior to this experiment; however, a musculoskeletal trained radiologist instructed them specifically in the ultrasound imaging and measurement of the dorsal Lisfranc ligament length. Both examiners were afforded the opportunity of multiple practice sessions prior to approval of their technical skills and procedures with respect to the imaging and measurement of the dorsal Lisfranc ligament length (as described below).

### Ultrasound technique

**Figure 4:**
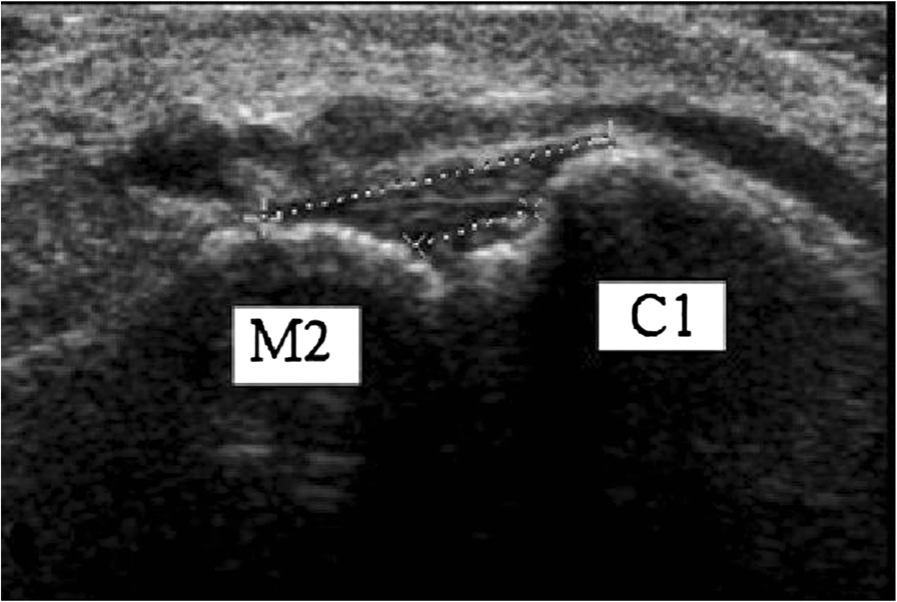
**Close-up sonographic image of the dorsal Lisfranc ligament.** C1 = Medial cuneiform, M2 = 2^nd^ metatarsal base, Dashed line with cross hairs indicate the boundaries of the dorsal Lisfranc ligament.

Subjects were seated in the calf raise apparatus with their knee and ankle relative joint angles at 90° and foot flat on the weight-bearing surface. Parallel lines were drawn on the dorsum of the foot in line with the dorsal Lisfranc ligament direction to ensure proper probe alignment. The ultrasound transducer was maintained in parallel orientation to the drawn lines throughout the procedure. Transmission gel was liberally applied directly to the skin superficial to the dorsal Lisfranc ligament for optimal transducer contact and signal penetration. Each randomly assigned stress/foot orientation condition was loaded prior to placing the transducer to the skin. Starting from the dorsomedial distal foot, the ultrasound probe was placed perpendicular to the skin surface, mid-shaft over the first metatarsal. The probe was moved proximally up the long axis of the first metatarsal in search for the first metatarsal-medial cuneiform joint congruence. Distal to the congruence, it was noted that the metatarsal base slopes toward the joint space which is marked by the hypoechoic synovial fluid. Proximal to the congruence, 0.5-1 centimeters onto the medial cuneiform, the probe was shifted laterally until the dorsomedial edge of the second metatarsal base came into view. The dorsal Lisfranc ligament lies within this area between the medial cuneiform and the second metatarsal base (Figure 4). This ultrasound imaging protocol was designed by the musculoskeletal trained radiologist and was used to train the examiners.

## Results

Fifty subjects (25 male and 25 female) participated in the study with an average age of 24.74 years (min 21; max 32), average weight of 75.3 Kg (min 44.1; max 119.5), and an average height of 173.9 cm (min 153.4; max 192.1). The average foot length was 25.4 cm (min 19; max 30), and the average foot width was 9.5 cm (min 8.0; max 11.5). The average length of the dorsal Lisfranc ligament at all conditions was 7.01 mm (min 4.16; max 11.36), which corresponds to 2.76% foot length and 7.38% foot width.

Our findings are arranged according to within-session intra-rater (Table 1), between-session intra-rater (Table 2), and between-session inter-rater (Table 3). The average reliability and the 95% confidence intervals are included for each condition (foot angle by stress load) separately. The average ICCs found are not consistent across conditions for the within-session intra-rater or the between-session intra/inter-rater measurements (Tables 1, 2 and 3). The ultrasound measurement of the dorsal Lisfranc ligament length was shown to be more consistent with the foot in the 15° abducted position and under higher stress load (Table 4, reflected in bold).

**Table 1:** Within-session intra-rater reliability ICC values (n = 100)

**Foot angle**	**Stress load**	**ICC**	**(95% CI)**
0°	Low	0.880	(0.838-0.914)
	Medium	0.884	(0.844-0.917)
	High	0.913	(0.881-0.938)
15° abducted	Low	0.873	(0.829-0.909)
	Medium	0.896	(0.858-0.925)
	High	0.885	(0.844-0.917)
	** Average**	**0.889**	

**Table 2:** Between-session intra-rater reliability ICC values (n = 40)

**Foot angle**	**Stress load**	**ICC**	**(95% CI)**
0°	Low	0.607	(0.368-0.771)
	Medium	0.778	(0.619-0.876)
	High	0.757	(0.585-0.864)
15° abducted	Low	0.797	(0.647-0.887)
	Medium	0.730	(0.544-0.847)
	High	0.811	(0.670-0.895)
	** Average**	**0.747**	

**Table 3:** Between-session inter-rater reliability ICC values (n = 40)

**Foot angle**	**Stress load**	**ICC**	**(95% CI)**
0°	Low	0.647	(0.423-0.796)
	Medium	0.638	(0.410-0.790)
	High	0.719	(0.528-0.841)
15° abducted	Low	0.691	(0.487-0.824)
	Medium	0.647	(0.423-0.796)
	High	0.776	(0.615-0.875)
	** Average**	**0.685**	

**Table 4:** Average ICCs by foot angle position and stress load

		**Within-session intra-rater reliability**	**Between-session intra-rater reliability**	**Between-session inter-rater reliability**
**Foot Angle**	**0°**	0.892	0.714	0.668
	**15° abducted**	0.885	**0.779**	**0.705**
**Stress Load**	**Low**	0.877	0.702	0.669
	**Medium**	0.890	0.754	0.643
	**High**	**0.899**	**0.784**	**0.748**

According to Fleiss et al., ICC values greater than 0.75 represent Excellent reliability, while 0.75 to 0.40 represent Good to Fair reliability and values less than 0.40 represent Poor reliability [16]. Landis et al. similarly describes ICC values from 0.81-1.00 as Almost Perfect, while ICCs of 0.61-0.80 show Substantial Strength of Agreement [17].

With respect to within-session intra-rater reliability (Table 1), the ICCs for all conditions are considered Almost Perfect according to Landis and considered well above the Excellent benchmark as defined by Fleiss. For between-session intra-rater reliability (Table 2), a majority of the ICCs fall within Landis’ Substantial Strength of Agreement category while one condition (high stress load at 15° abducted foot position) has an ICC 0.811 which is Almost Perfect. Finally, the between-session inter-rater reliability (Table 3) ICCs are within Landis’ Substantial Strength of Agreement category for all conditions. One condition (high stress load at 15° abducted foot position) reaches Fleiss’ Excellent standard with an ICC 0.776. The average overall ICCs decreased from within-session intra-rater to between-session intra-rater to between-session inter-rater (0.889, 0.747, and 0.685 respectively) (Tables 1, 2 and 3).

## Discussion

Prior to the development of any diagnostic imaging protocol intended for clinical use there is a need of a well-established, documented and reliable measurement technique that provides objective data for assessment purposes. This way clinicians can be assured that their results/measurements are meaningful. According to the aforementioned reliability scales [16,17], our data suggests that our methodology using ultrasound to image and measure the dorsal Lisfranc ligament length is convincingly reliable.

The measurement protocol we used seems extremely reproducible within a session, as shown by the within-session intra-rater ICCs. This reflects the consistency in measurement of the same individual within a single session and is well above the excellent mark. As expected, the reliability drops when there is at least 24 hours between ultrasound analyses. The between-session intra-rater ICCs reflect the consistency in measurements taken by a single examiner over two different sessions, and they show a decrease in reliability for each condition as compared to within a single session. Furthermore, the between-session inter-rater ICCs again show an overall decrease in reliability as compared to only one examiner. These differences, however, are small suggesting that similarly trained examiners can produce reliable results. We believe that the overall average ICCs using this novel ultrasound imaging and measurement protocol of the dorsal Lisfranc ligament length are remarkably high. Consideration must be taken that the examiners while having no formal musculoskeletal sonography training went through an extensive protocol specific training with a practicing musculoskeletal trained radiologist prior to participation in the current study. Our results suggest that clinicians with comprehensive knowledge of Lisfranc joint anatomy who undergo appropriate training in the use of ultrasound imaging technology and in the measurement of the dorsal Lisfranc ligament length will be able to duplicate reliably this ultrasound imaging/measurement protocol and obtain sonographic images of the dorsal Lisfranc ligament in a consistent manner.

It is interesting to note that the high stress load and 15° of foot abduction show the most reproducible measurements. Our data suggests that single leg standing with the foot 15° externally rotated provides a possible, clinically feasible position for Lisfranc ligament sonographic assessment. Similar to the radiographic protocols, patients presenting with acute pain from Lisfranc ligamentous injuries may not tolerate full weight-bearing, especially in an abducted position which appears to unlock the Lisfranc joint and renders it unstable [18]. A diagnostic nerve block could be administered prior to ultrasound evaluation to avoid patient guarding against weight-bearing. However, low and medium stress loads (representing non weight-bearing and bipedal stance, respectively) show substantial reliability.

Our findings suggest performing the dorsal Lisfranc ligament ultrasound imaging and measurement protocol with the patient’s foot at 15° externally rotated and at full weight-bearing on the affected limb to ensure the most reliable results. Weight bearing condition is consistent with the recommendations made for radiographic imaging of the Lisfranc joint [11]. Although it is not advocated to use ultrasound to replace standard radiographic imaging in a patient presenting with midfoot pain, it is believed that ultrasound imaging is a reliable adjunctive modality, especially when there is high clinical suspicion of a Lisfranc ligament strain injury despite negative radiographs.

Evaluation of Lisfranc joint pathology typically begins with conventional radiography; however, radiographs often show false-negative findings, and multiple studies have shown conventional radiographs to be unreliable in detecting Lisfranc ligamentous injuries [19]. While clinicians traditionally look for diastasis between the medial cuneiform and second metatarsal base of greater than 2 mm, a Lisfranc ligament injury may be associated with a diastasis less than this [6]. In some cases of Lisfranc ligament injury, the dorsal Lisfranc ligament may appear hypoechoic and edematous on ultrasound [6] while radiographs are negative. Although many clinicians are hesitant to recommend/use ultrasound imaging, it is less expensive, safer, and can be used in a more dynamic setting than both x-ray and MRI, making it a promising diagnostic technique for Lisfranc ligament pathology assessment.

The use of the seated calf raise apparatus, while afforded us the opportunity to consistently and accurately simulate physiologically/clinically feasible weight-bearing stress loads on the foot, is not a clinically available device. No musculoskeletal trained imaging technicians were involved in this experiment. The ultrasound examiners were podiatric medical students who had detailed knowledge of Lisfranc joint anatomy through a comprehensive lower extremity anatomy course and received extensive training from a practicing musculoskeletal trained radiologist specific to the imaging protocol used in the study. Specifically, the ultrasound training pertained to diagnostic ultrasound and its applications to soft tissue visualization with emphasis on the foot. The training lasted slightly longer than a month with regular meetings that amounted to an estimated minimum of 40 hours of practice. The inter-rater reliability protocol used in the current study was also between-session, and as such, it likely gave poorer inter-rater outcomes than is actually the case. The imaging/measurement of the dorsal Lisfranc ligament length was undertaken here as suggested by the literature to reflect the structure and potential pathology of the Lisfranc joint complex [6].

## Conclusions

The use of a novel ultrasound imaging and measurement protocol for the dorsal Lisfranc ligament length in asymptomatic subjects has been reported for both intra-rater and inter-rater reliability. It has been determined that the most reproducible images were obtained with the foot in 15° externally rotated orientation and full weight-bearing. Further studies are needed to assess the potential use of this protocol in the clinical setting and the use of ultrasound imaging and measurement of the dorsal Lisfranc ligament length as a diagnostic tool for patients with symptomatic midfoot pain. This imaging modality holds promise as a fast, inexpensive addition to the standard Lisfranc complex injury assessment.

## Competing interests

The authors declare that they have no competing interests.

## Authors’ contributions

DDR carried out data collection and drafting the manuscript. NCG carried out the literature review and drafting the manuscript. JJM carried out data collection and drafting the manuscript. KF carried out clinical guidance of the study and revising the manuscript. VV carried out the oversight, design, statistical analysis, and the multiple revisions of the manuscript. All authors have read and approved the final manuscript.

## Authors’ information

David D. Rettedal, BA, and Joshua J. Marshall, BA, are podiatric medical students at Des Moines University, College of Podiatric Medicine and Surgery, Des Moines, IA, USA.

Nathan C. Graves, DPM, is a former Des Moines University, CPMS student, currently a resident at Shands/University of Florida, Department of Orthopaedic Surgery, Jacksonville, FL, USA.

Katherine Frush, DPM, is an Assistant Professor at Des Moines University, College of Podiatric Medicine and Surgery, Des Moines, IA, USA.

Vassilios Vardaxis, PhD, is a Professor for the Department of Physical Therapy and the Director of Research for the College of Podiatric Medicine and Surgery, Des Moines University, Des Moines, IA, USA.
